# Long noncoding RNA PXN-AS1-L promotes non-small cell lung cancer progression via regulating PXN

**DOI:** 10.1186/s12935-019-0734-0

**Published:** 2019-01-22

**Authors:** Zhifa Zhang, Zhaohui Peng, Junying Cao, Jiaqi Wang, Yongyu Hao, Kai Song, Yan Wang, Wei Hu, Xuesong Zhang

**Affiliations:** 1 Department of Orthopaedic Surgery, the PLA General Hospital, Beijing, 100000 China; 2 Department of Radiology, Changzheng Hospital, Second Military Medical University, Shanghai, 200003 China; 3 Department of Radiology, Jinan Military General Hospital, Jinan, 250031 Shandong China; 4 Department of Ultrasonography, General Hospital of Shenyang Military Region, Shenyang, 110016 Liaoning China; 5 Department of Orthopaedic Surgery, China Medical University, Shenyang, 110001 Liaoning China; 6 Department of General Surgery, Changhai Hospital, Second Military Medical University, Shanghai, 200433 China

**Keywords:** Long noncoding RNA, Non-small-cell lung cancer, Proliferation, Migration, PXN

## Abstract

**Background:**

Increasingly evidences suggest that long noncoding RNAs (lncRNAs) play important roles in various cancers. LncRNA PXN-AS1-L is recently revealed to act as on oncogene in liver cancer. However, the expression, functions, and mechanisms of action of PXN-AS-L in non-small cell lung cancer (NSCLC) remain unclear.

**Methods:**

The expression of PXN-AS1-L in primary NSCLC tissues, NSCLC bone metastasis tissues, and cell lines was measured by quantitative real-time PCR. The correlations between PXN-AS1-L expression and clinicopathological characteristics of NSCLC patients were analyzed by Pearson Chi square test and log-rank test. The roles of PXN-AS1-L in cell viability, proliferation, apoptosis, and migration of NSCLC cells, and in vivo NSCLC tumor growth were investigated by a series of gain-of-function and loss-of-function assays. The regulatory roles of PXN-AS1-L on PXN were determined by quantitative real-time PCR and western blot.

**Results:**

PXN-AS1-L was up-regulated in NSCLC tissues compared with noncancerous lung tissues, and PXN-AS1-L was further up-regulated in NSCLC bone metastasis tissues. Increased expression of PXN-AS1-L was positively associated with advanced TNM stages and poor prognosis. Gain-of-function and loss-of-function assays showed that PXN-AS1-L increased cell viability, promoted cell proliferation, inhibited cell apoptosis, and promoted cell migration of NSCLC cells. Xenograft assays showed that PXN-AS1-L also promoted NSCLC tumor growth in vivo. Mechanistically, we found that PXN-AS1-L, as an antisense transcript of PXN, up-regulated the expression of PXN. PXN was also up-regulated in NSCLC tissues. The expression of PXN and PXN-AS1-L was positively correlated in NSCLC tissues. Furthermore, PXN knockdown attenuated the roles of PXN-AS1-L in increasing cell viability, promoting cell proliferation, inhibiting cell apoptosis, and promoting cell migration of NSCLC cells.

**Conclusions:**

Our data revealed that PXN-AS1-L is up-regulated and acts as an oncogene in NSCLC via up-regulating PXN. Our data suggested that PXN-AS1-L might serve as a potential prognostic biomarker and therapeutic target for NSCLC.

## Background

Lung cancer is the most common malignancy and the leading cause of cancer death around the world, with an estimated 2,093,876 new cases and 1,761,007 deaths in 2018 worldwide [1]. The major histological subtype of lung cancer is non-small cell lung cancer (NSCLC), which mainly includes adenocarcinoma and squamous cell carcinoma, and accounts for approximate 80% of lung cancers [2]. Except the NSCLC patients which are diagnosed at early stages and could be cured by surgical resection, most NSCLC patients still have poor outcomes with the 5-year survival rate of about 20% [3]. Thus, it is of paramount importance to reveal the underlying molecular mechanisms contributing to the tumorigenesis and progression of NSCLC in order to develop novel therapeutic strategies for NSCLC [4–6].

Accumulating evidences have revealed that most of human genome is transcribed, but only about 2% of human genome encode for proteins [7]. Therefore, most of human transcripts don’t encode for proteins. Among these non-coding transcripts, long noncoding RNAs (lncRNAs) have gradually attracted people’s attention [8–12]. LncRNA is a class of non-coding transcripts with more than 200 nucleotides in length and limited protein coding potential [13–15]. Many lncRNAs are revealed to play important roles in various pathophysiological processes [16–19]. As to tumors, lncRNAs have been reported to regulate almost every aspect of biological behaviors of cancer cells, including cell proliferation, apoptosis, senescence, autophagy, migration, invasion, and so on [20–25]. Furthermore, many lncRNAs are dysregulated in various cancers and associated with early diagnosis and/or prognosis [26–28].

Several lncRNAs have been reported to have oncogenic or tumor suppressing roles in NSCLC. LncRNA MALAT1 is a positive marker of lung cancer metastasis [29]. LncRNA PVT1 is reported to promote NSCLC cell proliferation [30]. LncRNA BANCR is reported to promote NSCLC metastasis [31]. LncRNA MIR22HG is reported to inhibit cell survival [32]. LncRNA P53RRA is reported to promote ferroptosis and apoptosis [33]. LncRNA MUC5B-AS1 is reported to promote metastasis [34]. LncRNA linc00460 is reported to promote cell migration [35]. LncRNA LINC00473 is reported to promote lung cancer tumor growth [36]. Although the expression and function of these lncRNAs are studied in NSCLC, most lncRNAs transcribed from human genome are functionally unclear in NSCLC [37]. LncRNA PXN-AS1-L is a recently identified lncRNA, which is upregulated in hepatocellular carcinoma (HCC) and promoted HCC tumorigenesis via up-regulating PXN [38]. However, the expression, roles, and mechanisms of action of lncRNA PXN-AS1-L in NSCLC are still unknown.

In this study, we aimed to elucidate the expression pattern of lncRNA PXN-AS1-L in NSCLC via measuring PXN-AS1-L expression in noncancerous lung tissues, NSCLC tissues, NSCLC bone metastases tissues, normal bronchial epithelial cell line, and human NSCLC cell lines. We also investigated the biological functions of PXN-AS1-L in NSCLC using in vitro and in vivo gain-of-function and loss-of-function assays. Moreover, we explored the molecular mechanisms mediating the roles of PXN-AS1-L in NSCLC.

## Methods

### Cell culture and treatment

The human normal bronchial epithelial cell line 16HBE, human NSCLC cell lines NCI-H1975, A549, NCI-H1299, and SK-MES-1 were obtained from Cell Bank of Type Culture Collection of the Chinese Academy of Sciences (Shanghai, China). 16HBE cells were cultured in Dulbecco’s Modified Eagle’s Medium (Invitrogen, Carlsbad, CA, USA). NCI-H1975 and NCI-H1299 cells were cultured in RPMI-1640 Medium (Invitrogen). A549 cells were cultured in F-12K Medium (Invitrogen). SK-MES-1 cells were cultured in Eagle’s Minimum Essential Medium (Invitrogen). All the cells were maintained in the above described medium supplemented with 10% fetal bovine serum (Invitrogen) in a humidified incubator at 37 °C with 5% CO_2_. Where indicated, NSCLC cells were treated with 50 µM α-amanitin (Sigma-Aldrich, Saint Louis, MO, USA) for 0–24 h as shown in the article.

### Human tissue specimens

Sixty-six pairs of NSCLC tissues and adjacent noncancerous lung tissues, and ten NSCLC bone metastases tissues were acquired from NSCLC patients who underwent surgery at the General Hospital of Chinese People’s Liberation Army (Beijing, China). All the tissues were diagnosed and histologically confirmed by two pathologists. The resected specimens were immediately frozen in liquid nitrogen and stored at − 80 °C until use. The study was approved by the ethics committee of the General Hospital of Chinese People’s Liberation Army (Beijing, China) and written informed consent was obtained from all patients.

### Plasmids construction and transfection

PXN-AS1-L overexpression plasmid pcDNA3.1-PXN-AS1-L was constructed as previously described [38]. Briefly, PXN-AS1-L full-length transcript was PCR amplified by Thermo Scientific Phusion Flash High-Fidelity PCR Master Mix (Thermo-Fisher Scientific, Waltham, MA, USA) and subcloned into the *Bam*H I and *Eco*R V sites of pcDNA3.1 plasmid (Invitrogen). The primers sequences were: 5′-GGTACCGAGCTCGGATCCTCGCGTTGGAGGAGCTTG-3′ (forward) and 5′-GCCACTGTGCTGGATATCCTACAAAAAAAATTTATTTAATAAAA-3′ (reverse). The cDNA oligonucleotides suppressing PXN-AS1-L or PXN expression were designed and synthesized by GenePharma (Shanghai, China). After annealing, double strand oligonucleotides were inserted to the SuperSilencing shRNA expression plasmid pGPU6/Neo (GenePharma). A scrambled shRNA was used as a negative control and designated as shControl. The shRNA sequences were: for shPXN, 5′-CACCCCTGACGAAAGAGAAGCCTAATTCAAGAGATTAGGCTTCTCTTTCGTCAGGTTTTTTG-3′ (sense), 5′-GATCCAAAAAACCTGACGAAAGAGAAGCCTAATCTCTTGAATTAGGCTTCTCTTTCGTCAGG-3′ (anti-sense); for shRXN-AS1-L, 5′-CACCGCCCAGAGGAAATCAACAAGATTCAAGAGATCTTGTTGATTTCCTCTGGGCTTTTTTG-3′ (sense), 5′-GATCCAAAAAAGCCCAGAGGAAATCAACAAGATCTCTTGAATCTTGTTGATTTCCTCTGGGC-3′ (anti-sense); for shControl, 5′-CACCGTTCTCCGAACGTGTCACGTTTCAAGAGAACGTGACACGTTCGGAGAATTTTTTG-3′ (sense), 5′-GATCCAAAAAATTCTCCGAACGTGTCACGTTCTCTTGAAACGTGACACGTTCGGAGAAC-3′ (anti-sense). Transient transfection was performed using Lipofectamine 3000 (Invitrogen) in accordance with the manufacturer’s instruction.

### Construction of stable cell lines

To constructing PXN-AS1-L stably overexpressed A549 cells, pcDNA3.1-PXN-AS1-L or pcDNA3.1 was transfected into A549 cells and selected with neomycin (800 µg/ml) for 4 weeks. To constructing PXN-AS1-L stably depleted NCI-H1299 cells, shPXN-AS1-L or shControl was transfected into NCI-H1299 cells and selected with neomycin (1000 µg/ml) for 4 weeks.

### RNA extraction and quantitative real-time PCR analysis (qPCR)

Total RNA was extracted from indicted tissues or cells using TRIzol Reagent (Invitrogen) in accordance with the manufacturer’s protocol. Reverse transcription was performed using the M-MLV Reverse Transcriptase (Invitrogen) in accordance with the manufacturer’s protocol. Quantitative real-time PCR (qPCR) was performed in the StepOnePlus Real-Time PCR System (Applied Biosystems, Foster City, CA, USA) using the SYBR^®^ Premix Ex Taq™ II kit (Takara, Dalian, China) in accordance with the manufacturer’s protocols. β-actin was used as an endogenous control. The primers sequences were: for PXN-AS1-L, 5′-ACCCATCCTCAACTACCCC-3′ (forward) and 5′-ACTTCGTCTGTGCCTTCTGC-3′ (reverse); for PXN, 5′-TATCTCAGCCCTCAACACGC-3′ (forward) and 5′-GGCAGAAGGCACAGACGAA-3′ (reverse); for β-actin, 5′-GGGAAATCGTGCGTGACATTAAG-3′ (forward) and 5′-TGTGTTGGCGTACAGGTCTTTG-3′ (reverse). The relative expression of RNAs was calculated using the comparative Ct method.

### Western blot analysis

Total proteins were extracted using RIPA buffer (Beyotime, Shanghai, China). Identical quantities of proteins were separated by sodium dodecyl sulfate–polyacrylamide gel electrophoresis and transferred onto nitrocellulose filter membranes. After being blocked with 5% not-fat milk, the membranes were incubated with primary antibodies specific for PXN (Abcam, Hong Kong, China) or β-actin (Proteintech, Rosemont, IL, USA). After being washed, the membranes were incubated with IRdye 700-conjugated goat anti-mouse IgG or IRdye 800-conjugated goat anti-rabbit IgG and were detected using an Odyssey infrared scanner (Li-Cor, Lincoln, NE, USA).

### Analysis of cell proliferation and apoptosis

Cell proliferation was detected using Glo cell viability assay and Ethynyl deoxyuridine (EdU) incorporation assay. For Glo cell viability assay, 3000 indicated NSCLC cells were seeded each well in 96-well plate. At indicated time, cell viability was detected using the CellTiter-Glo Luminescent Cell Viability Assay (Promega, Madison, WI, USA) in accordance with the manufacturer’s protocol. EdU incorporation assay was carried out using the EdU kit (Roche, Mannheim, Germany) in accordance with the manufacturer’s protocol. The results were acquired and quantified with the Zeiss AxioPhot Photomicroscope (Carl Zeiss, Oberkochen, Germany) based on at least ten random fields. Cell apoptosis was detected using terminal deoxynucleotidyl transferase (TdT)-mediated dUTP nick end labeling (TUNEL) assay. Indicated NSCLC cells were treated with 25 ng/ml doxorubicin (Selleck, Houston, TX, USA) for 24 h. Then, cell apoptosis was detected using the TUNEL Cell Apoptosis Detection Kit (Beyotime) in accordance with the manufacturer’s protocol.

### Cell migration assay

Cell migration was detected using transwell assay. 5 × 10^4^ indicated NSCLC cells in serum-free medium were plated in the top chamber a 24-well transwell chamber (BD Biosciences, San Jose, CA, USA). Complete medium containing 10% fetal bovine serum was placed into the lower chamber. After incubation for 24 h, cells remaining on the top chamber were wiped off using a cotton swab, and the cells that had traversed the membranes were stained by crystal violet and counted.

### Nude mouse xenograft assays

3 × 10^6^ indicted NSCLC cells were subcutaneously injected into 6-week-old athymic BALB/c nude mice purchased from the Shanghai Experimental Animal Center of Chinese Academy of Sciences (Shanghai, China). Subcutaneous xenograft growth was measured weekly with a caliper, and the tumor volume was calculated as a × b^2^ × 0.5 (a, longest diameter; b, shortest diameter). The animal studies were approved by the ethics committee of the General Hospital of Chinese People’s Liberation Army (Beijing, China).

### Immunohistochemistry (IHC)

For IHC, the subcutaneous xenografts were formalin-fixed, paraffin-embedded, and cut into 4 µm sections. The sections were incubated with primary antibodies specific for Ki67 (Abcam) or cleaved caspase-3 (Cell Signaling Technology, Boston, MA, USA). After being washed, the sections were incubated with horseradish peroxidase-conjugated second antibody (Abcam) and visualized using DAB Horseradish Peroxidase Color Development Kit (Beyotime).

### Statistical analysis

All statistical analyses were performed using the GraphPad Prism Software. For comparisons, Student’s *t* test (two-sided), Wilcoxon signed-rank test, Mann–Whitney test, Pearson Chi square test, Log-rank test, and Pearson correlation analysis were performed as indicated. *P* values < 0.05 were considered as statistically significant.

## Results

### PXN-AS1-L was up-regulated in NSCLC and associated with poor prognosis

**Fig. 1:**
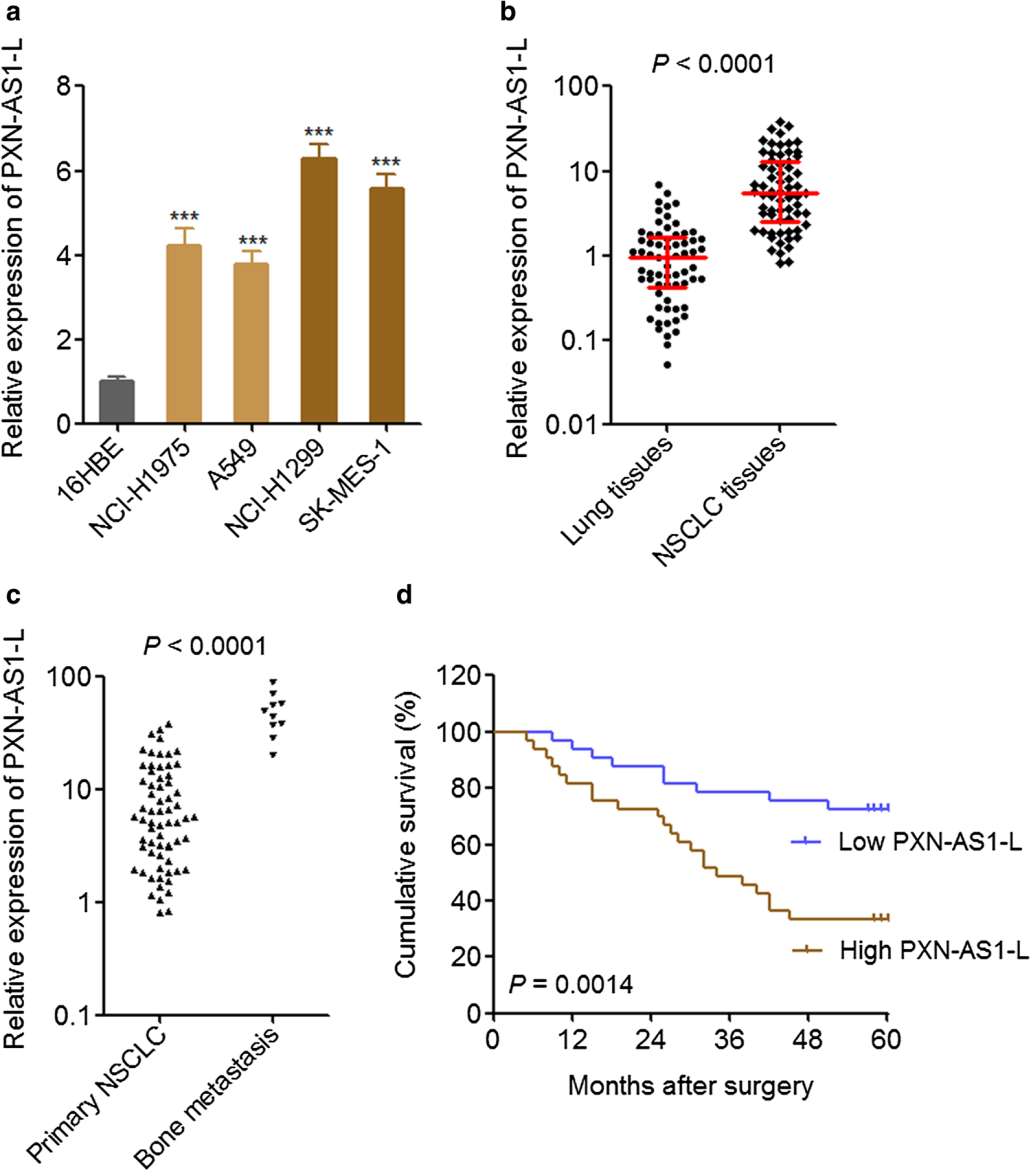
PXN-AS1-L was up-regulated in NSCLC and associated with poor prognosis. **a** The expressions of PXN-AS1-L in normal bronchial epithelial cell line 16HBE and NSCLC cell lines NCI-H1975, A549, NCI-H1299, and SK-MES-1 were detected by qPCR. Results are shown as mean ± SD of three independent experiments. ***P < 0.001 by Student’s *t*-test. **b** The expressions of PXN-AS1-L in 66 pairs of NSCLC tissues and adjacent noncancerous lung tissues were detected by qPCR. *P *< 0.0001 by Wilcoxon signed-rank test. **c** The expressions of PXN-AS1-L in 66 primary NSCLC tissues and 10 NSCLC bone metastases tissues were detected by qPCR. *P *< 0.0001 by Mann–Whitney test. **d** Kaplan–Meier survival analysis of the correlation between PXN-AS1-L expression and overall survival of these 66 NSCLC patients. The median PXN-AS1-L expression level was used as the cut-off. *P *< 0.0001 by Log-rank test

To investigate the expression pattern of PXN-AS1-L in NSCLC, we first measured the expression of PXN-AS1-L in normal bronchial epithelial cell line 16HBE and NSCLC cell lines NCI-H1975, A549, NCI-H1299, SK-MES-1. The results displayed that PXN-AS1-L was significantly up-regulated in NSCLC cell lines compared with that in normal bronchial epithelial cell line, and further up-regulated in NSCLC cell lines derived from metastatic sites (NCI-H1299 and SK-MES-1) (Fig. 1a). Then, we collected 66 pairs of NSCLC tissues and adjacent noncancerous lung tissues and measured the expression of PXN-AS1-L in these tissues. The results displayed that the expression of PXN-AS1-L was significantly higher in NSCLC tissues than that in adjacent noncancerous lung tissues (Fig. 1b). Furthermore, we collected 10 NSCLC bone metastases tissues and also measured the expression of PXN-AS1-L. The results displayed that the expression of PXN-AS1-L was further higher in bone metastases tissues than that in primary NSCLC tissues (Fig. 1c).

The correlations between the expression of PXN-AS1-L in NSCLC tissues and clinicopathological characteristics of these 66 NSCLC patients were analyzed. As shown in Table 1, increased expression of PXN-AS1-L was positively associated with larger tumor size, positive lymph nodes metastasis, and advanced TNM stages, but not associated with age, gender, and histologic subtype. Kaplan–Meier survival analysis displayed that NSCLC patients with higher PXN-AS1-L expression had worse survival than those with lower PXN-AS1-L expression (Fig. 1d). All these data together demonstrated that PXN-AS1-L was up-regulated in NSCLC, further up-regulated in metastatic NSCLC cells and tissues. Increased expression of PXN-AS1-L predicted poor outcome of NSCLC patients.

**Table 1:** Correlation between PXN-AS1-L expression and clinicopathological characteristics of NSCLC patients

Variables	Number	PXN-AS1-L		*P* value*
		Low	High^a^	
All cases	66	33	33	
Age				0.620
≥ 60	37	20	17	
< 60	29	13	16	
Gender				1.000
Male	48	24	24	
Female	18	9	9	
Histologic subtype				1.000
Squamous cell carcinoma	30	15	15	
Adenocarcinoma	36	18	18	
Tumor size (cm)				*0.021*
≤ 3	11	9	2	
> 3	55	24	31	
Lymph nodes metastasis				*0.003*
Positive	26	7	19	
Negative	40	26	14	
TNM stage				*0.000*
I	28	23	5	
II	27	10	17	
III	11	0	11	

^a^ The median expression level of PXN-AS1-L was used as the cut-off

* *P* value was acquired by Pearson Chi square test

### PXN-AS1-L overexpression promoted NSCLC cell proliferation and migration

**Fig. 2:**
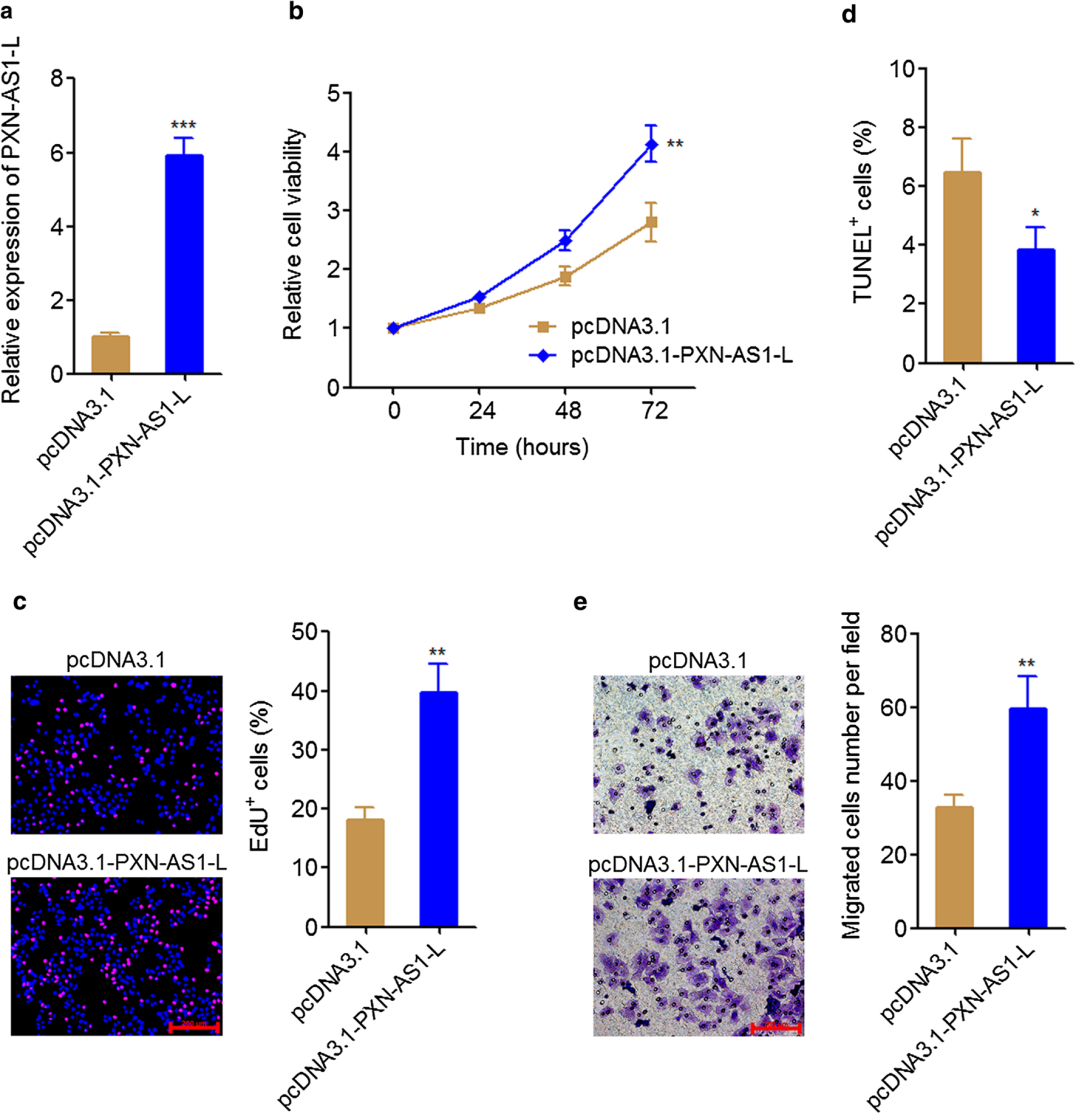
PXN-AS1-L overexpression promoted NSCLC cell proliferation and migration. **a** The expressions of PXN-AS1-L in PXN-AS1-L stably overexpressed and control A549 cells were detected by qPCR. **b** Cell viability of PXN-AS1-L stably overexpressed and control A549 cells was detected by Glo cell viability assays. **c** Cell proliferation of PXN-AS1-L stably overexpressed and control A549 cells was detected by EdU incorporation assays. The red color indicates EdU-positive cells. Scale bars = 200 μm. **d** Cell apoptosis of PXN-AS1-L stably overexpressed and control A549 cells was detected by TUNEL assays. **e** Cell migration of PXN-AS1-L stably overexpressed and control A549 cells was detected by transwell assays. Scale bars = 100 μm. Results are shown as mean ± SD of three independent experiments. *P < 0.05, **P < 0.01, ***P < 0.001 by Student’s *t*-test

To reveal the biological effects of PXN-AS1-L on NSCLC, we stably overexpressed PXN-AS1-L in A549 cells which has a relative low expression of PXN-AS1-L among NSCLC cell lines by transfecting PXN-AS1-L overexpression plasmid (Fig. 2a). Glo cell viability assays displayed that PXN-AS1-L overexpression increased cell viability of A549 cells (Fig. 2b). EdU incorporation assays also displayed that PXN-AS1-L overexpression promoted cell proliferation of A549 cells (Fig. 2c). TUNEL assays displayed that PXN-AS1-L overexpression inhibited cell apoptosis of A549 cells (Fig. 2d). Transwell assays displayed that PXN-AS1-L overexpression promoted cell migration of A549 cells (Fig. 2e). All these data together demonstrated that PXN-AS1-L overexpression promoted cell proliferation, inhibited cell apoptosis, and promoted cell migration of NSCLC cells, suggesting that PXN-AS1-L has oncogenic roles in NSCLC.

### PXN-AS1-L knockdown reduced NSCLC cell proliferation and migration

**Fig. 3:**
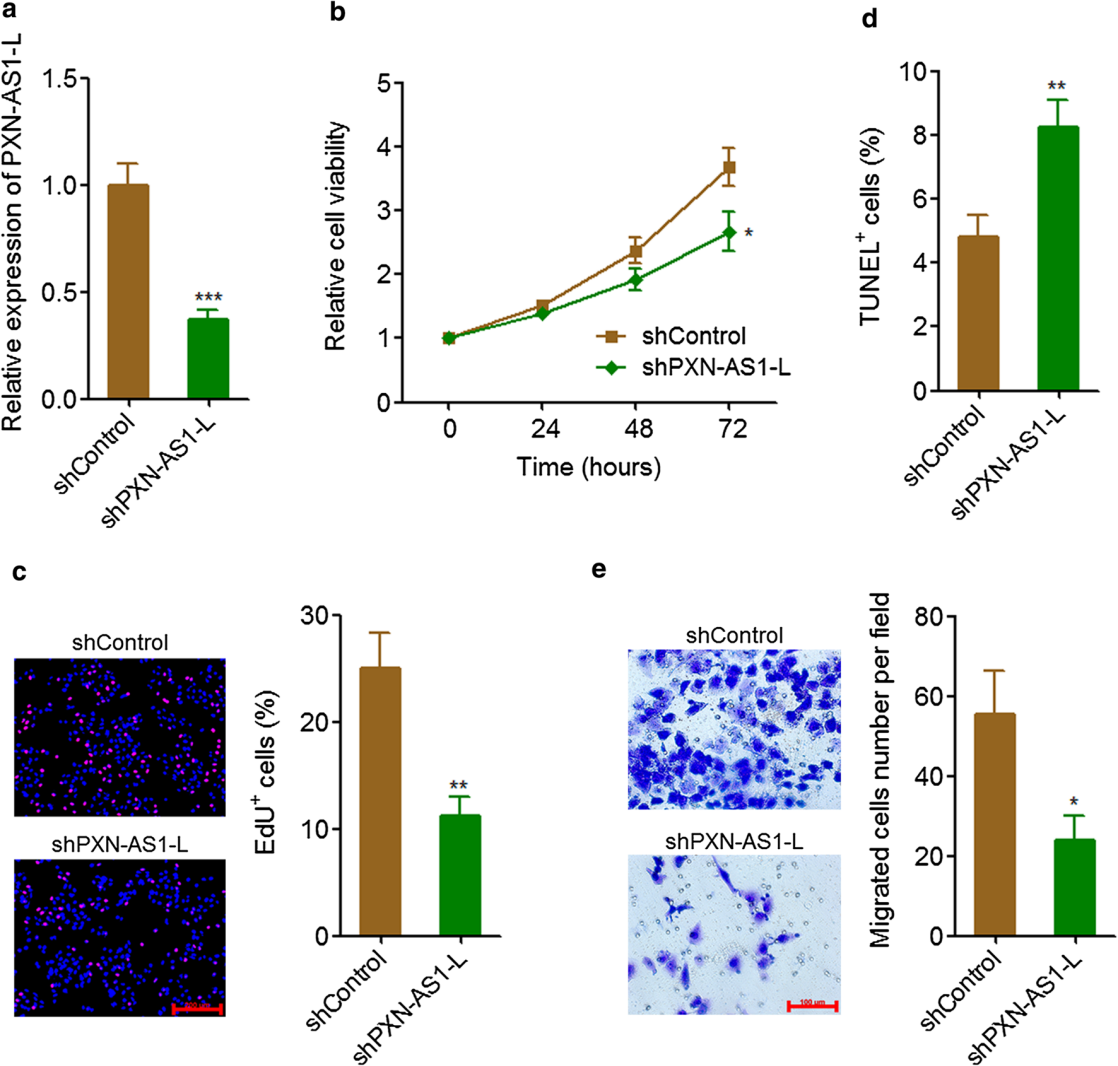
PXN-AS1-L knockdown inhibited NSCLC cell proliferation and migration. **a** The expressions of PXN-AS1-L in PXN-AS1-L stably knocked down and control NCI-H1299 cells were detected by qPCR. **b** Cell viability of PXN-AS1-L stably knocked down and control NCI-H1299 cells was detected by Glo cell viability assays. **c** Cell proliferation of PXN-AS1-L stably knocked down and control NCI-H1299 cells was detected by EdU incorporation assays. The red color indicates EdU-positive cells. Scale bars = 200 μm. **d** Cell apoptosis of PXN-AS1-L stably knocked down and control NCI-H1299 cells was detected by TUNEL assays. **e** Cell migration of PXN-AS1-L stably knocked down and control NCI-H1299 cells was detected by transwell assays. Scale bars = 100 μm. Results are shown as mean ± SD of three independent experiments. *P < 0.05, **P < 0.01, ***P < 0.001 by Student’s *t*-test

To further confirm the oncogenic roles of PXN-AS1-L on NSCLC, we stably knocked down PXN-AS1-L expression in NCI-H1299 cells which has a relative high expression of PXN-AS1-L among NSCLC cell lines by transfecting PXN-AS1-L specific shRNA (Fig. 3a). Glo cell viability assays displayed that PXN-AS1-L knockdown reduced cell viability of NCI-H1299 cells (Fig. 3b). EdU incorporation assays also displayed that PXN-AS1-L knockdown inhibited cell proliferation of NCI-H1299 cells (Fig. 3c). TUNEL assays displayed that PXN-AS1-L knockdown promoted cell apoptosis of NCI-H1299 cells (Fig. 3d). Transwell assays displayed that PXN-AS1-L knockdown inhibited cell migration of NCI-H1299 cells (Fig. 3e). All these data together demonstrated that PXN-AS1-L knockdown inhibited cell proliferation, promoted cell apoptosis, and inhibited cell migration of NSCLC cells, supporting the oncogenic roles of PXN-AS1-L in NSCLC.

### PXN-AS1-L promoted the growth of NSCLC xenograft in vivo

**Fig. 4:**
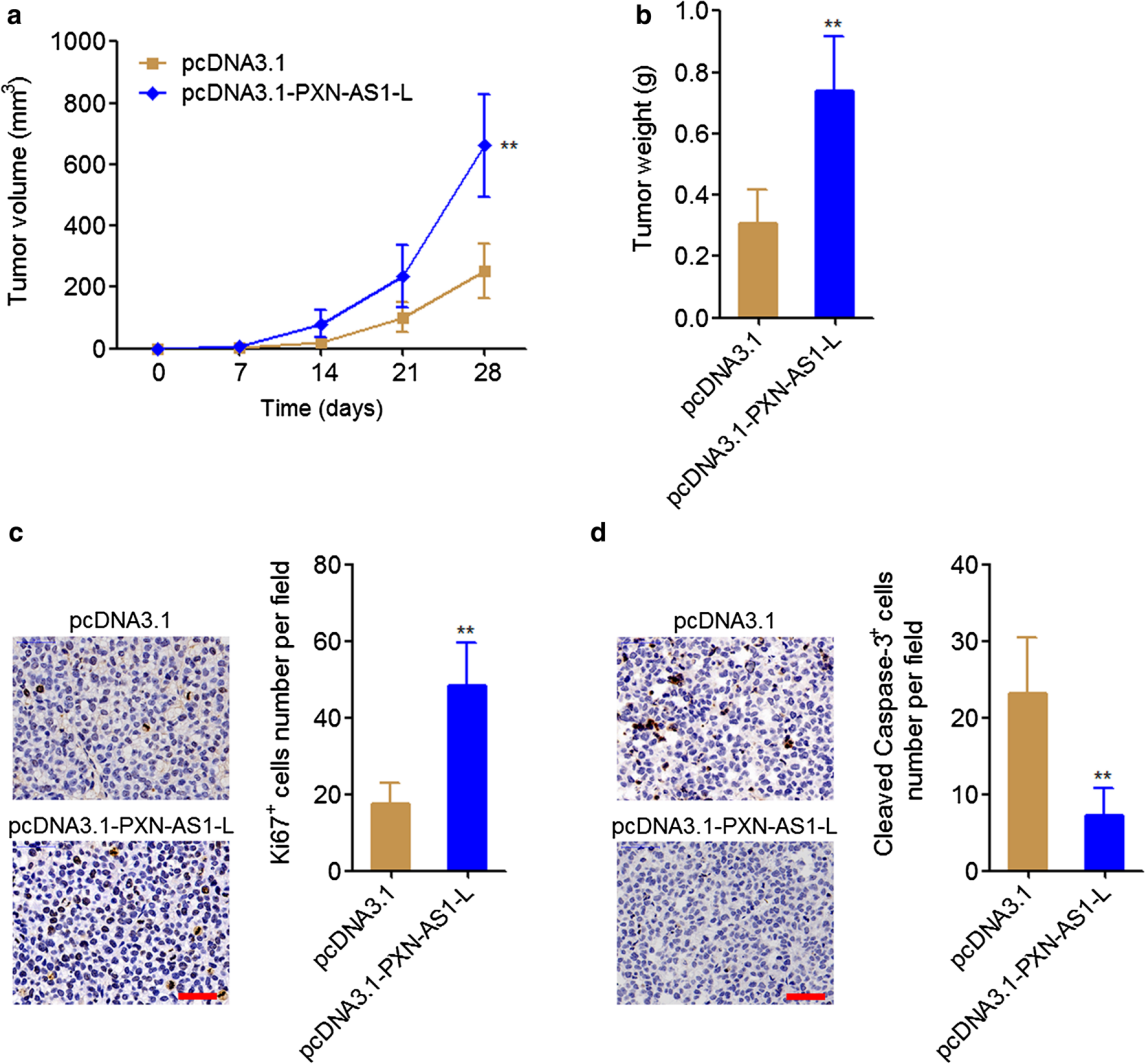
PXN-AS1-L promoted NSCLC xenograft growth in vivo. **a** Tumor volumes of subcutaneous xenografts derived from PXN-AS1-L stably overexpressed and control A549 cells were measured every 7 days. **b** Tumor weights of subcutaneous xenografts derived from PXN-AS1-L stably overexpressed and control A549 cells at the 28th day after injection. **c** In vivo cell proliferation of PXN-AS1-L stably overexpressed and control A549 cells was evaluated using Ki67 immunohistochemistry staining of subcutaneous xenografts. Scale bars = 50 µm. **d** In vivo cell apoptosis of PXN-AS1-L stably overexpressed and control A549 cells was evaluated using cleaved caspase-3 immunohistochemistry staining of subcutaneous xenografts. Scale bars = 50 µm. Results are shown as mean ± SD of six mice in each group. **P < 0.01 by Mann–Whitney test

To explore whether PXN-AS1-L also have oncogenic roles in vivo, PXN-AS1-L stably overexpressed and control A549 cells were subcutaneously injected into nude mice. Xenografts growth rates were measured every 7 days. At the 28th day, the xenografts were excised and weighed. The results displayed that PXN-AS1-L overexpression markedly promoted xenograft growth in vivo, and PXN-AS1-L overexpressed A549 cells formed much larger tumor than those formed by control A549 cells (Fig. 4a, b). Ki67 staining of xenograft displayed that PXN-AS1-L overexpression promoted A549 cell proliferation in vivo (Fig. 4c). Cleaved caspase-3 staining of xenograft displayed that PXN-AS1-L overexpression inhibited A549 cell apoptosis in vivo (Fig. 4d). All these data together demonstrated that PXN-AS1-L also had oncogenic roles in vivo.

### PXN-AS1-L up-regulated PXN expression

**Fig. 5:**
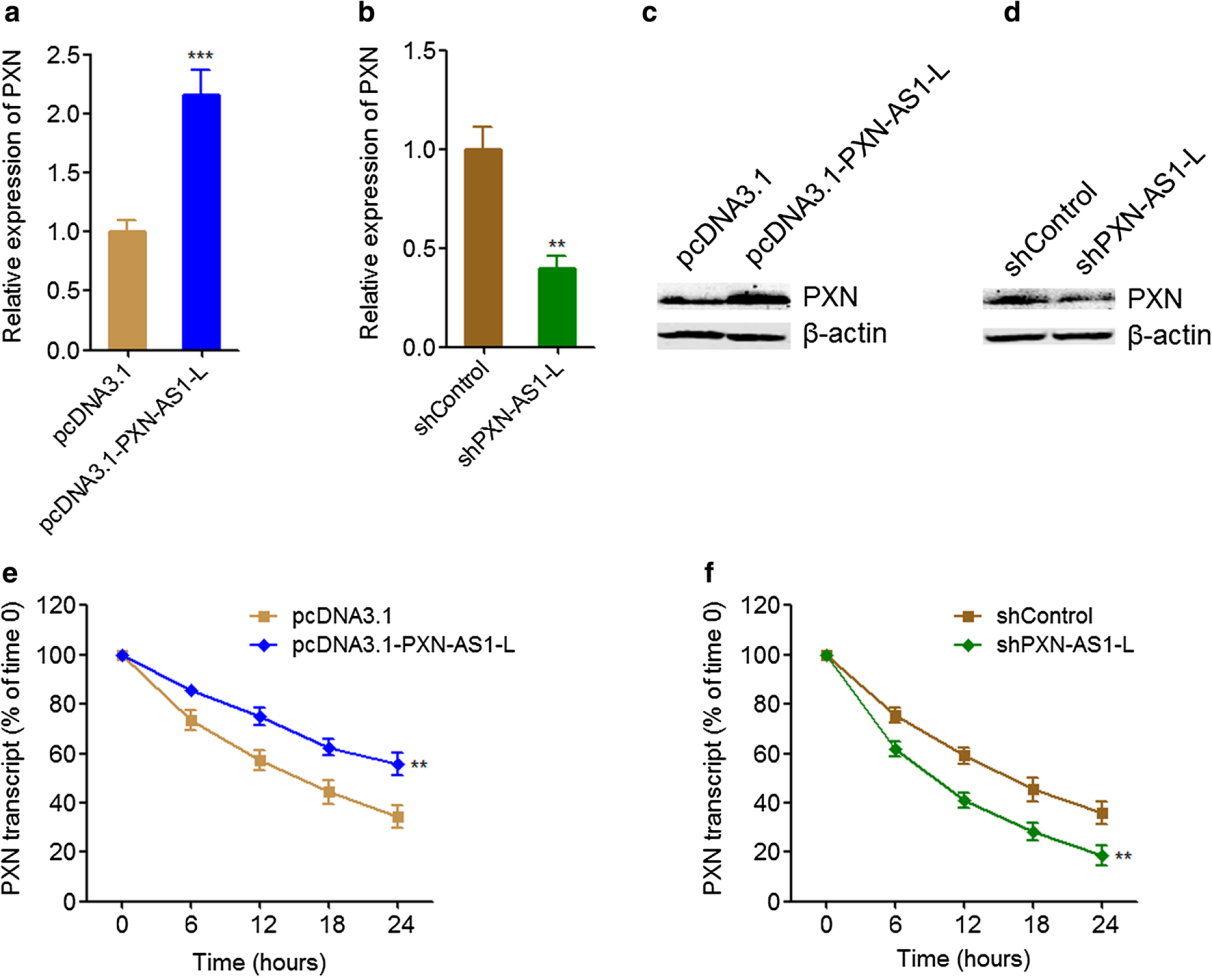
PXN-AS1-L up-regulated PXN expression. **a** PXN mRNA levels in PXN-AS1-L stably overexpressed and control A549 cells were detected by qPCR. **b** PXN mRNA levels in PXN-AS1-L stably depleted and control NCI-H1299 cells were detected by qPCR. **c** PXN protein levels in PXN-AS1-L stably overexpressed and control A549 cells were detected by western blot. **d** PXN protein levels in PXN-AS1-L stably depleted and control NCI-H1299 cells were detected by western blot. **e** After transient overexpressing PXN-AS1-L in A549 cells, the stability of PXN mRNA over time was determined after blocking new RNA synthesis with α-amanitin (50 µM) and normalized to 18S rRNA (a product of RNA polymerase I that is unchanged by α-amanitin). **f** After transient depleting PXN-AS1-L in NCI-H1299 cells, the stability of PXN mRNA over time was determined after blocking new RNA synthesis with α-amanitin (50 µM) and normalized to 18S rRNA. Results are shown as mean ± SD of three independent experiments. **P < 0.01, ***P < 0.001 by Student’s *t*-test

PXN-AS1-L is previously reported to up-regulate PXN expression in HCC cells [38]. PXN has been revealed to play oncogenic roles in NSCLC in several reports [39–41]. Therefore, we first investigated whether PXN-AS1-L also regulates PXN expression in NSCLC and whether the oncogenic roles of PXN-AS1-L in NSCLC are dependent on the regulation of PXN. The expressions of PXN in PXN-AS1-L stably overexpressed and control A549 cells, and PXN-AS1-L stably depleted and control NCI-H1299 cells were measured by qRT-PCR and western blot. The results displayed that PXN-AS1-L overexpression up-regulated PXN mRNA level (Fig. 5a). Conversely, PXN-AS1-L knockdown reduced PXN mRNA level (Fig. 5b). Western blot assays also displayed that PXN-AS1-L overexpression up-regulated PXN protein level and while PXN-AS1-L knockdown reduced PXN protein level (Fig. 5c, d). PXN-AS1-L is reported to decrease PXN mRNA degradation and increase PXN mRNA stability in HCC cells [38]. To investigate whether the same mechanism was employed by PXN-AS1-L in NSCLC, we next determined the effects of PXN-AS1-L on PXN mRNA stability in NSCLC cell. After transiently overexpressing PXN-AS1-L in A549 cells or depleting PXN-AS1-L in NCI-H1299 cells, the cells were treated with α-amanitin to block new RNA synthesis. Next, the loss of PXN mRNA was measured. As shown in Fig. 5e, f, PXN-AS1-L overexpression elongated the half-life of PXN mRNA, and conversely, PXN-AS1-L knockdown shortened the half-life of PXN mRNA, which suggested that PXN-AS1-L also increase PXN mRNA stability in NSCLC cells. All these data together demonstrated that PXN-AS1-L up-regulated PXN expression in NSCLC.

### PXN was up-regulated in NSCLC and positively correlated with PXN-AS1-L expression

**Fig. 6:**
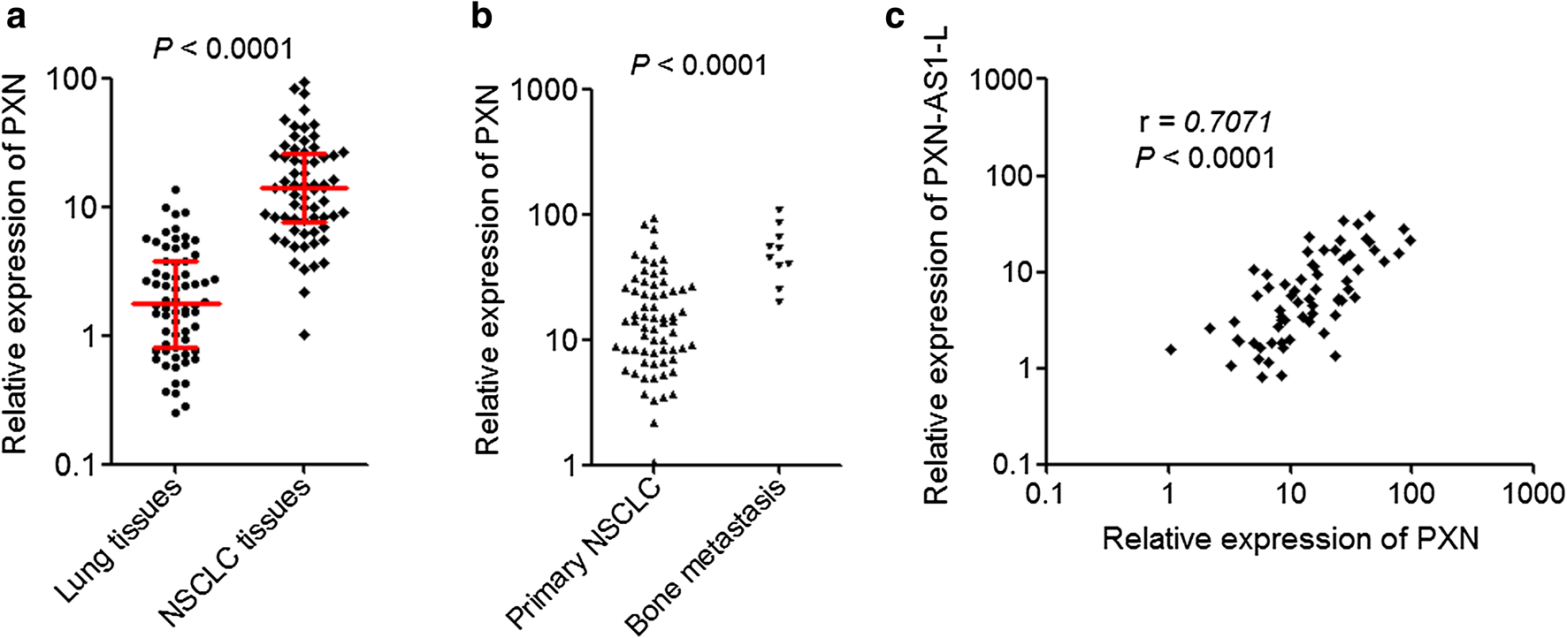
PXN was up-regulated in NSCLC and positively correlated with PXN-AS1-L expression. **a** The expressions of PXN in 66 pairs of NSCLC tissues and adjacent noncancerous lung tissues were detected by qPCR. *P *< 0.0001 by Wilcoxon signed-rank test. **b** The expressions of PXN-AS1-L in 66 primary NSCLC tissues and 10 NSCLC bone metastases tissues were detected by qPCR. *P* < 0.0001 by Mann–Whitney test. **c** The correlation between the expression level of PXN-AS1-L and PXN was analyzed in these 66 NSCLC tissues. r = 0.7071, *P *< 0.0001 by Pearson correlation analysis

To explore whether the regulation of PXN by PXN-AS1-L also exist in vivo, we measured PXN expression in the same 66 pairs of NSCLC tissues and adjacent noncancerous lung tissues used in Fig. 1b. The results displayed that the expression of PXN was also significantly higher in NSCLC tissues than that in adjacent noncancerous lung tissues (Fig. 6a). Furthermore, our results also displayed that the expression of PXN was further higher in bone metastases tissues than that in primary NSCLC tissues (Fig. 6b). Correlation analysis displayed that the expression level of PXN-AS1-L was positively correlated with that of PXN in these 66 NSCLC tissues (Fig. 6c), supporting the positive regulation of PXN by PXN-AS1-L.

### PXN knockdown attenuated the oncogenic roles of PXN-AS1-L in NSCLC

**Fig. 7:**
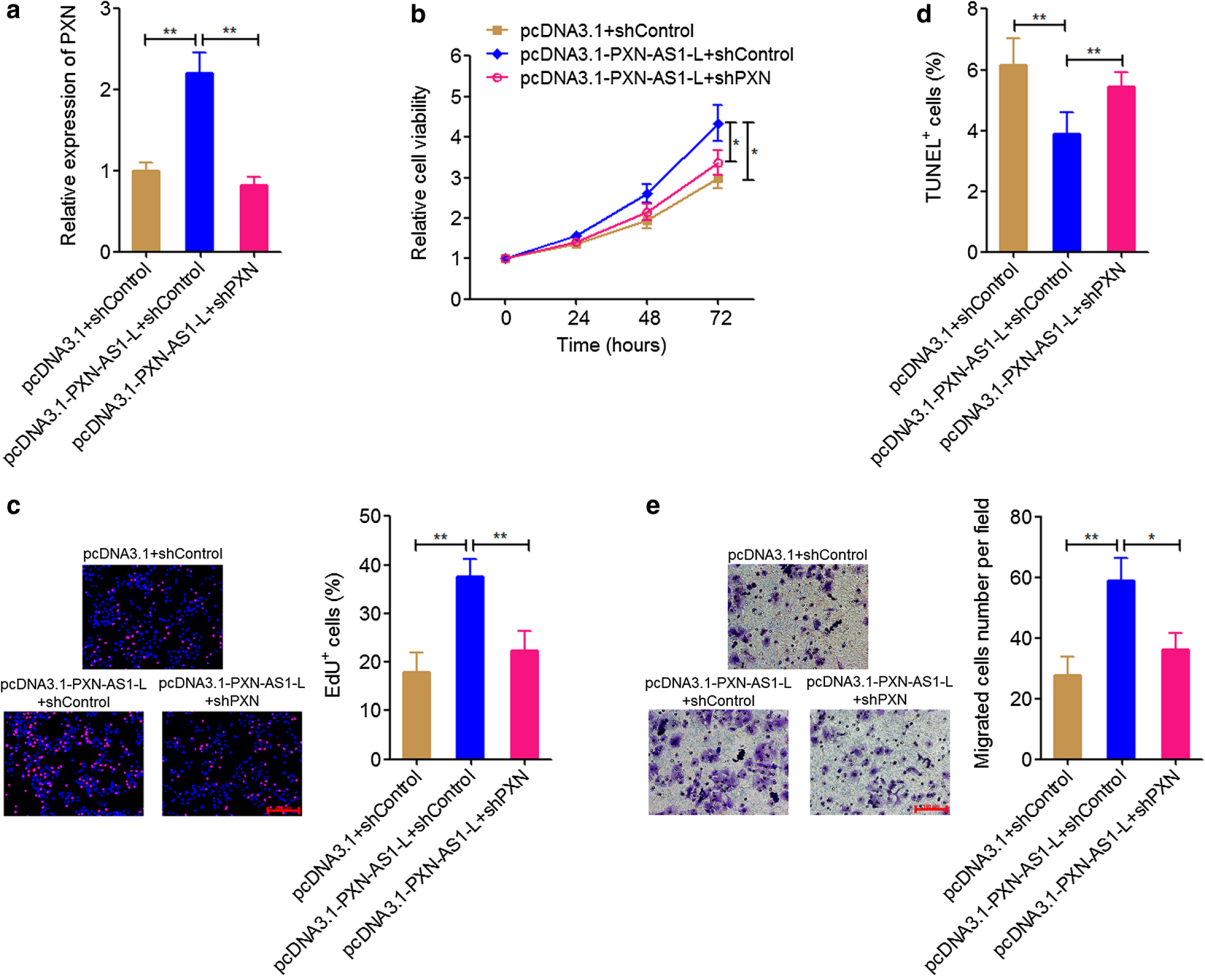
PXN knockdown attenuated the oncogenic roles of PXN-AS1-L in NSCLC. **a** The expressions of PXN in PXN-AS1-L stably overexpressed and control A549 cells after transient transfection of PXN specific shRNA were detected by qPCR. **b** Cell viability of PXN-AS1-L stably overexpressed and control A549 cells after transient transfection of PXN specific shRNA was detected by Glo cell viability assays. **c** Cell proliferation of PXN-AS1-L stably overexpressed and control A549 cells after transient transfection of PXN specific shRNA was detected by EdU incorporation assays. The red color indicates EdU-positive cells. Scale bars = 200 μm. **d** Cell apoptosis of PXN-AS1-L stably overexpressed and control A549 cells after transient transfection of PXN specific shRNA was detected by TUNEL assays. **e** Cell migration of PXN-AS1-L stably overexpressed and control A549 cells after transient transfection of PXN specific shRNA was detected by transwell assays. Scale bars = 100 μm. Results are shown as mean ± SD of three independent experiments. *P < 0.05, **P < 0.01 by Student’s *t*-test

To explore whether the oncogenic roles of PXN-AS1-L in NSCLC are dependent on the positive regulation of PXN, we knocked down PXN in PXN-AS1-L stably overexpressed A549 cells by transient transfection of PXN specific shRNA (Fig. 7a). Glo cell viability assays displayed that PXN knockdown attenuated the increasing of cell viability induced by PXN-AS1-L overexpression (Fig. 7b). EdU incorporation assays also displayed that PXN knockdown attenuated the pro-proliferative roles of PXN-AS1-L (Fig. 7c). TUNEL assays displayed that PXN knockdown attenuated the cell apoptosis repressive roles of PXN-AS1-L (Fig. 7d). Transwell assays displayed that PXN knockdown attenuated the increasing of cell migration induced by PXN-AS1-L overexpression (Fig. 7e). All these data together demonstrated that PXN knockdown attenuated the roles of PXN-AS1-L in promoting cell proliferation, inhibiting cell apoptosis, and promoting cell migration of NSCLC cells. These data also suggested that the oncogenic roles of PXN-AS1-L in NSCLC were at least partially dependent on the positive regulation of PXN.

## Discussion

LncRNA PXN-AS1-L has 863 nucleotides in length. The gene encoding PXN-AS1-L locates at chromosome 12q24.23 and is reverse complementary to *PXN*. PXN-AS1-L is recently identified to have oncogenic roles in HCC [38]. In this study, we further studied the expression, roles, and mechanisms of action of PXN-AS1-L in NSCLC.

First, we found that PXN-AS1-L is up-regulated in NSCLC cell lines compared with normal bronchial epithelial cell line. Moreover, PXN-AS1-L is further up-regulated in NSCLC cell lines derived from metastatic sites (NCI-H1299 and SK-MES-1) compared with that derived from primary sites (NCI-H1975 and A549). In clinical tissues specimens, we also found that PXN-AS1-L is up-regulated in NSCLC tissues compared with noncancerous lung tissues and is further up-regulated in NSCLC bone metastases tissues. Analyses of the correlation between PXN-AS1-L expression and clinicopathological characteristics revealed that PXN-AS1-L was positively associated with tumor size, lymph nodes metastasis, advanced TNM stages, and poor prognosis. These data confirmed that PXN-AS1-L is up-regulated in NSCLC and implied that PXN-AS1-L may be involved in the progression of NSCLC. Whether the up-regulation of PXN-AS1-L in cancer is lung and liver cancer specific or cancer-popular need further investigation. Furthermore, multi-center prospective study investigating the correlation between the expression of PXN-AS1-L and prognosis of NSCLC patients would be more significant for the application of PXN-AS1-L for clinical outcome prediction.

Second, we investigated the in vitro and in vivo roles of PXN-AS1-L in NSCLC using gain-of-function and loss-of-function assays. Our results revealed that PXN-AS1-L overexpression increases NSCLC cell viability, promotes NSCLC cell proliferation, inhibits NSCLC cell apoptosis, and promotes NSCLC cell migration. Conversely, PXN-AS1-L knockdown decreases NSCLC cell viability, inhibits NSCLC cell proliferation, promotes NSCLC cell apoptosis, and represses NSCLC cell migration. Therefore, our data demonstrated that PXN-AS1-L also acts as an oncogene in NSCLC, similar to the roles of PXN-AS1-L in HCC. Whether the oncogenic role of PXN-AS1-L is lung and liver cancer specific or cancer-popular also need further investigation. Nevertheless, the up-regulation of PXN-AS1-L and oncogenic roles of PXN-AS1-L in NSCLC suggested that PXN-AS1-L may be a potential therapeutic target for NSCLC. Increasing evidences have shown that noncoding RNAs could be targeted by chemically modified complementary oligonucleotides, which are revealed to be effective treatment in animal models and clinical trials involving humans [42, 43]. Therefore, developing chemically modified complementary oligonucleotides targeting PXN-AS1-L would be potential therapeutic strategy for NSCLC.

The focal adhesion protein PXN mediates critical signal transduction and plays important roles in cell survival and migration [44, 45]. Due to the reverse complementation between *PXN*-*AS1*-*L* and *PXN*, we investigated whether PXN-AS1-L regulates PXN and whether PXN is the mediator of the oncogenic roles of PXN-AS1-L in NSCLC. In this study, we found that PXN-AS1-L up-regulated PXN expression. Similar to the expression pattern of PXN-AS1-L in NSCLC, PXN is also up-regulated in NSCLC tissues compared with noncancerous lung tissues and is further up-regulated in NSCLC bone metastases tissues. The expression of PXN-AS1-L is positively associated with that of PXN in NSCLC tissues. Furthermore, knockdown of PXN attenuated the oncogenic roles of PXN-AS1-L in NSCLC. All these data support the positive regulation of PXN by PXN-AS1-L and the importance of PXN in the oncogenic roles of PXN-AS1-L in NSCLC.

## Conclusions

In conclusion, our data showed that PXN-AS1-L is up-regulated in NSCLC, predicts poor outcome of NSCLC patients, and has oncogenic roles in NSCLC via up-regulating PXN. Our data also implied that PXN-AS1-L may be a potential prognostic biomarker and therapeutic target for NSCLC.

## Abbreviations

NSCLC: non-small cell lung cancer
lncRNAs: long noncoding RNAs
HCC: hepatocellular carcinoma
qPCR: quantitative real-time PCR
EdU: ethynyl deoxyuridine
TUNEL: terminal deoxynucleotidyl transferase (TdT)-mediated dUTP nick end labeling
IHC: immunohistochemistry
